# Intention-driven ankle exoskeleton training acutely alters swing foot clearance distribution: a preliminary study

**DOI:** 10.3389/fnbot.2026.1912825

**Published:** 2026-08-05

**Authors:** Hanatsu Nagano, Rezaul Begg, Eri Sarashina, Hiroaki Kawamoto

**Affiliations:** 1 Institute of Systems and Information Engineering, University of Tsukuba, Tsukuba, Japan; 2 Institute for Health and Sport (IHES), Victoria University, Footscray, VIC, Australia

**Keywords:** ankle exoskeleton, fall prevention and control, foot clearance, motor control, neurorehabilitation, surface electromyography signal

## Abstract

**Introduction:**

Tripping is a leading cause of falls, and Minimum Foot Clearance (MFC)—the lowest vertical swing-foot displacement during mid-swing—is a key gait event associated with tripping risk. Preventing tripping requires not only sufficiently high MFC but also consistent control across steps, which depends on neuromotor rather than strength-based mechanisms. We hypothesized that aligning movement intention with repeated, optimal ankle motion using a surface electromyography (sEMG)-driven exoskeleton would improve neuromotor consistency and MFC characteristics.

**Methods:**

A total of 12 healthy adults completed 5 min of treadmill walking at 4 km/h before and after a 5-min ankle exoskeleton training sequence (‘feet-flat, toes-up, feet-flat, heels-up’, 60 bpm). The Vicon motion capture system tracked the reflective markers placed on the heel and toe to collect MFC dataset. The exoskeleton (i.e., Hybrid Assistive Limb) detects voluntary neural drive through sEMG and converts it into assisted ankle motion, supporting intention-based neuromotor training.

**Results:**

The obtained MFC dataset showed that training increased the lowest percentile of the MFC distribution (i.e., the risky tail) by 0.39 cm, reduced step-to-step variability, and increased dataset skewness, suggesting reduction of both low and high extremes in swing-foot clearance.

**Discussion:**

These preliminary findings suggest that brief intention-based ankle exoskeleton training may acutely improve safety-relevant characteristics of MFC control. Further studies in older adults and neurological populations are required to determine clinical relevance for tripping-risk reduction.

## 1 Introduction

**Figure 1:**
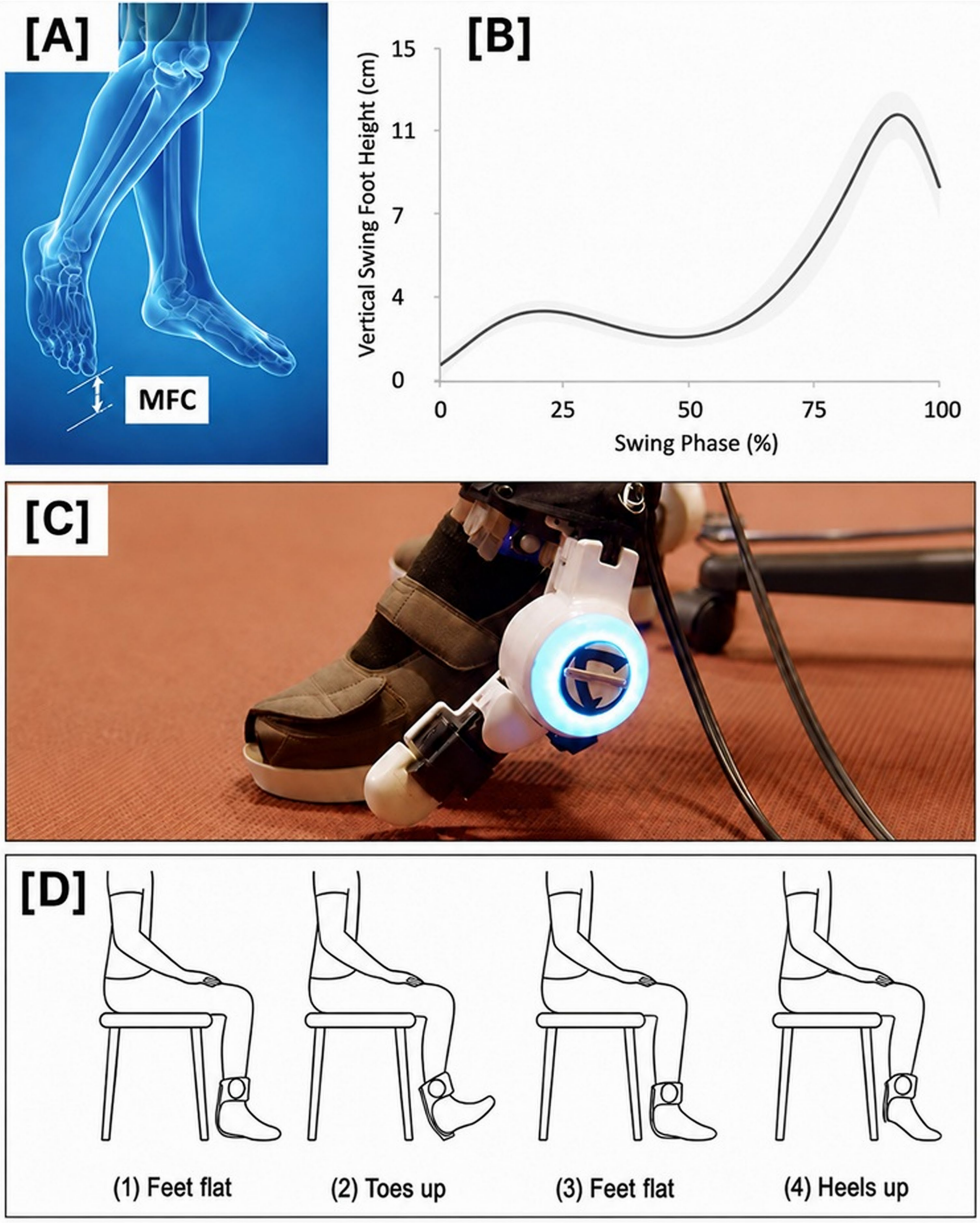
**(A)** Minimum Foot Clearance (MFC) (Nagano, 2022); **(B)** swing foot clearance and MFC (Nagano, 2022); **(C)** ankle exoskeleton attached to the participant; **(D)** ankle exoskeleton training sequence: (i) feet flat, (ii) toes up, (iii) feet flat, and (iv) heels up.

Falls-related injuries are a serious concern for vulnerable populations, contributing to high injury rates and substantial medical costs (Florence et al., 2018; World Health Organization (WHO), 2021). Among various contributing factors, tripping is the leading cause, accounting for up to 53% of all fall incidents (Berg et al., 1997; Blake et al., 1988). Understanding the mechanisms behind tripping and developing targeted interventions is therefore essential for risk reduction. Tripping is defined as unexpected contact between the swing foot and a walking surface or object, creating momentum that destabilizes the walker. During the gait cycle, Minimum Foot Clearance (MFC)—the point of minimal vertical toe displacement in the mid-swing phase—has been identified as the critical event associated with high risk of tripping falls, due to (i) very low foot clearance, (ii) near maximum swing foot speed—a cause of a significant impact if tripping occurs, and (iii) suboptimal foot positioning for maintaining balance (Winter, 1992) (Figures 1A,B).

Safe swing-foot control can be defined as maintaining sufficiently high MFC across multiple steps (Begg et al., 2007). The primary contributor to elevating MFC is ankle dorsiflexion, which plays a greater role than other lower-limb joint motions (Moosabhoy and Gard, 2006). However, excessive dorsiflexion is unlikely to be necessary, as unseen obstacles are generally of low height, requiring only subtle adjustments in swing-foot elevation. Tripping prevention can be, therefore, summarized as providing minimum yet sufficient MFC ‘constantly’ during locomotion. Thus, simply achieving high MFC does not warrant safe swing foot clearance, but the previous studies (Begg et al., 2007; Best and Begg, 2008; Mills et al., 2008) agreed on the importance of consistent MFC control over multiple gait cycles, as it is the occasional unpredicted and less controlled low foot clearance that poses the tripping risk.

The ability to replicate consistent walking patterns is governed by neuromotor control rather than strength alone (Brach et al., 2015; Patel et al., 2021), necessitating targeted approaches to enhance neuromotor function. One successful intervention involves real-time biofeedback training, designed to maintain high and consistent MFC control during treadmill walking through visual feedback (Nagano et al., 2020; Tirosh et al., 2013). In brief, vertical swing toe displacement is projected on a monitor in real-time, with two horizontal lines indicating the target MFC range, allowing treadmill walkers to practice consistent swing foot clearance.

While this biofeedback protocol has shown positive effects for post-stroke individuals (Nagano et al., 2020), it required specialized equipment (e.g., treadmill, monitor, safety harness, and motion capture system) and oversight by physiotherapists and technicians, making it less feasible in busy everyday rehabilitation settings. In addition, this biofeedback module attempts to guide toe-position control without directly engaging the underlying neuromuscular circuits. To address this limitation, another approach involves an active exoskeleton system that detects efferent neural signals transmitted from the central nervous system to the target muscle groups and assists the wearer in replicating the intended movements (Kawamoto and Sankai, 2005). When impairments exist in accurately conveying neural commands, such a system can capture efferent activity through surface electromyography (sEMG) electrodes and reproduce the corresponding motion. It can be hypothesized that neuromotor functions, including the consistency of movement patterns, can be optimized by aligning the neural inputs with the intended motor outputs precisely (Morishita and Inoue, 2016).

Powered ankle exoskeletons and orthoses have been widely investigated as direct methods to assist swing-phase dorsiflexion and improve foot clearance during walking. Previous systems have used different control strategies, including proportional myoelectric control (Kao and Ferris, 2009), gait-event detection with angular-velocity control (Nakagawa et al., 2022), and impedance-based or hybrid torque–impedance assistance (Pruyn et al., 2025), to provide dorsiflexion assistance during the swing phase. These studies indicate that powered ankle assistance can increase foot clearance during device-assisted walking. However, most of these approaches aim to provide real-time assistance during gait.

In contrast to these gait-assistance devices, the ankle exoskeleton used in the present study was developed to support neuromotor training for ankle movement control rather than to provide continuous assistance during walking (Kubota et al., 2022).

As shown in Figures 1C, this device assists repetitive dorsiflexion–plantarflexion movements (Kubota et al., 2022) and has been reported to improve symptoms such as foot drop in individuals with peroneal nerve palsy. As ankle dorsiflexion-plantarflexion is key to gait mechanics including foot clearance (Moosabhoy and Gard, 2006), this study investigates whether repetitive exercise utilizing the active exoskeleton improves MFC characteristics particularly movement consistency.

Successful reduction of MFC variability is an important outcome for preventing tripping falls; however, it is ultimately attributable to minimizing the occurrence of occasional low-clearance events. To investigate this, analytical approaches should be shifted toward focusing on extremely, and often exceptionally, low MFC values. Instead of reporting only central tendency measures such as the mean or median, the first percentile (1%) value was examined, representing the lowest MFC observed in roughly every 100 steps. Previous studies have indeed reported significant differences in the lowest MFC percentiles between healthy individuals and stroke survivors, despite similar mean values (Nagano et al., 2022b).

In the current study, a comprehensive analytical framework was thus employed, integrating conventional metrics (central tendency, dispersion), dataset distribution characteristics (skewness, kurtosis), and lower-end percentile analysis (1%). We hypothesized that ankle-focused neuromotor training with an assistive exoskeleton would improve the consistency of swing-foot clearance. Then, the reduction in MFC variability could be attributed to the suppression of both higher and lower extremes in the MFC distribution. These hypotheses were tested by comparing gait patterns before and after a brief ankle-assist exoskeleton intervention during treadmill walking.

## 2 Methods

### 2.1 Participants

A total of 12 healthy adults were recruited from university volunteers for this study. Participants were required to meet the following inclusion criteria: being healthy, able to walk on a treadmill for 30 min without a break, and free from past or present injuries that could affect their walking patterns. The entire experimental protocol was explained to participants, and informed consent, as approved by the Victoria University Human Research Ethics Committee, was voluntarily signed prior to participation.

### 2.2 Protocol

Gait testing was conducted for 5 min on a treadmill (AMTI) at 4 km/h before and after a 5-min exoskeleton training session. Data collection started only after the treadmill belt had reached the target speed of 4 km/h; therefore, the acceleration phase was not included in the analysis. The Vicon Bonita system (Nexus), equipped with 12 cameras operating at 200 Hz, was used to track reflective markers placed on key foot segment landmarks, including the heel (proximal end of the shoe) and toe (anterior superior surface of the shoe). To determine MFC, toe-off and heel-contact events were identified using standard kinematic conventions (Nagano et al., 2022b). MFC was defined as the local minimum vertical displacement of the toe during the mid-swing phase (Figures 1A,B; Begg et al., 2007). When a clear local minimum was absent, maximum horizontal velocity was used to define MFC (Nagano et al., 2022b). Prior to analysis, raw position data were processed with a low-pass Butterworth filter (6 Hz), and any occluded data lasting up to 0.1 s were interpolated. When marker occlusion exceeded 0.1 s or could not be reliably interpolated, the affected step and the corresponding paired contralateral step were removed from the analysis.

### 2.3 Ankle exoskeleton

An ankle-assist exoskeleton (Hybrid Assistive Limb; HAL, Cyberdyne Inc., Japan) was used for dorsiflexion–plantarflexion training. The system detects voluntary motor intent from surface electromyographic (sEMG) signals recorded over the tibialis anterior and soleus muscles and generates proportional torque assistance that reflects the user’s intended joint motion. Although the manufacturer refers to these inputs as “bioelectrical signals,” the control input is fundamentally sEMG corresponding to voluntary neural drive. The internal controller processes these signals to extract the onset and amplitude characteristics of muscle activation, allowing the device to reproduce ankle motion in synchrony with the user’s motor intent. This intention-based interactive mechanism promotes active engagement rather than passive actuation.

The dual-joint ankle module (Figure 1D) was designed to support functional control and rehabilitation in individuals with impaired foot motion, such as foot drop. In this study, the same system was applied to healthy adults to examine its potential effects on neuromechanical regulation of swing-foot clearance.

### 2.4 Training methods

As illustrated in Figure 1D, the ankle exoskeletons were attached bilaterally, and participants performed a repetitive ankle-movement protocol in a seated position, consisting of four sequential actions: (1) feet flat, (2) toes up, (3) feet flat, and (4) heels up, as previously described (Nagano et al., 2022a). Each movement phase was synchronized to a metronome set at 60 beats per minute to maintain consistent pacing.

The protocol was based on standard exoskeleton-assisted ankle training principles, designed to activate both dorsiflexor and plantarflexor muscle groups alternately. An assistance gain level of 20 (from the available 0 to 1,200 range) was selected to provide minimal augmentation while ensuring participants maintained voluntary effort, following the previous pilot-study (Nagano et al., 2022a). Higher gain levels are typically used for individuals with impaired neural drive (e.g., spinal cord injury or stroke), whereas a lower level was deemed appropriate for healthy adults. HAL setup took approximately 10 min while the participant remained seated. Training lasted for 5 min and was immediately followed by a 20-min seated interval before the post-training gait assessment. This interval was included to allow removal of the HAL and surface electrodes and to minimize the immediate influence of fatigue, warm-up, and residual effects of device use on the post-training gait assessment.

### 2.5 Parameters

**Figure 2:**
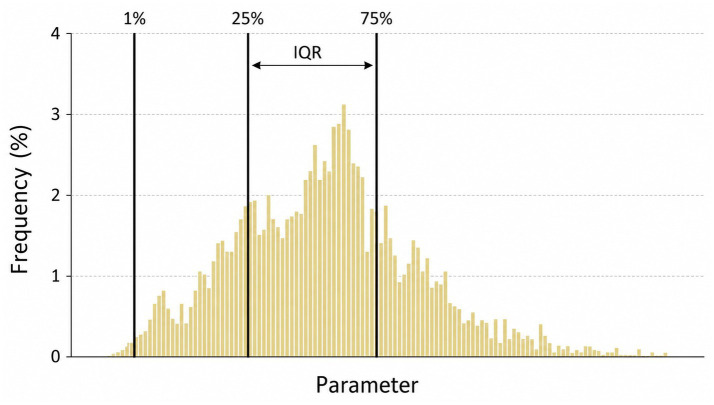
Histogram description of the first percentile (1%) indicating the lowest part of the MFC dataset; IQR defined between 25th and 75th percentiles [adapted].

In the current study, MFC characteristics were described by central tendency (i.e., mean, median), step-to-step variability [i.e., standard deviation (SD), interquartile range (IQR)], the lowest part of the dataset [i.e., 1st percentile (1%)], dataset distribution (i.e., skewness and kurtosis). Details of these dependent variables were further described in the previous studies (Begg et al., 2007; Nagano et al., 2022b). As visualized in Figure 2, 1st percentile (1%) indicates the lowest part of the MFC dataset, while IQR is the variability measure between 25 and 75%. Our recent study (Nagano et al., 2022b) started focusing on the lowest part of the dataset, which is likely to reflect the unintentional tripping risk rather than central tendency measures.

### 2.6 Statistical analysis

A 2 × 2 (training × limb) multivariate analysis of variance (MANOVA) was conducted to examine the main effects and interaction of the 5-min ankle training across the two limbs. Statistical significance was accepted at *p* < 0.05. Outcome measures were MFC distribution metrics, including the mean, median, standard deviation (SD), interquartile range (IQR), 1st percentile, skewness and kurtosis. Partial eta-squared (*ηp*^2^) values were computed as effect-size estimates for the multivariate and follow-up univariate effects, and were interpreted using conventional thresholds: 0.01 as small, 0.06 as medium, and 0.14 as large.

## 3 Results

Table 1 summarizes the Minimum Foot Clearance (MFC) characteristics before (Baseline) and after the (Post) intervention. The significant overall effects (*F*_7,38_ = 3.864, *p* = 0.003, *ηp*^2^ = 0.416) indicated that a 5-min ankle training changed MFC characteristics.

**Table 1:** MFC characteristics for Baseline and Post training.

Statistical descriptor	Baseline		Post		Statistical effects (*p*-value)		
	Right	Left	Right	Left	Training	Limb	Training × limb
Mean (cm)	2.36	1.97	2.14	1.99	0.677	0.258	0.596
SD (cm)	0.43	0.46	0.32	0.34	**0.015**	0.626	0.864
Median (cm)	2.35	1.94	2.12	1.97	0.653	0.232	0.582
IQR (cm)	0.53	0.54	0.42	0.44	0.090	0.846	0.965
1% (cm)	1.14	0.90	1.48	1.34	**0.033**	0.295	0.780
Skew	−0.24	−0.41	0.27	0.53	**0.020**	0.899	0.479
Kurt	2.81	7.34	11.52	1.13	0.831	0.615	0.205

Bold values indicate statistically significant effects (*p* < 0.05).

For neuromotor control aspects, standard deviation (SD) was reduced by 0.12 cm following training (*F*_1,44_ = 6.469, *p* = 0.015, *ηp*^2^ = 0.128). Another measure of variability, the interquartile range (IQR), exhibited a similar trend, although it did not reach statistical significance (*p* = 0.090).

There were no changes in central tendency measures (mean, median) in MFC height, but the first percentile (1%) increased by 0.39 cm after training (*F*_1,44_ = 4.838, *p* = 0.033, *ηp*^2^ = 0.099). The average skewness score across individuals was significantly higher in the Post condition (*F*_1,44_ = 5.868, *p* = 0.020, *ηp*^2^ = 0.118). No significant effects were found for the main limb comparisons, nor were there any training × limb interaction effects. Although kurtosis changed numerically between limbs and conditions, no significant training, limb, or training × limb interaction effect was detected; therefore, it was not interpreted as a systematic training-related change.

**Figure 3:**
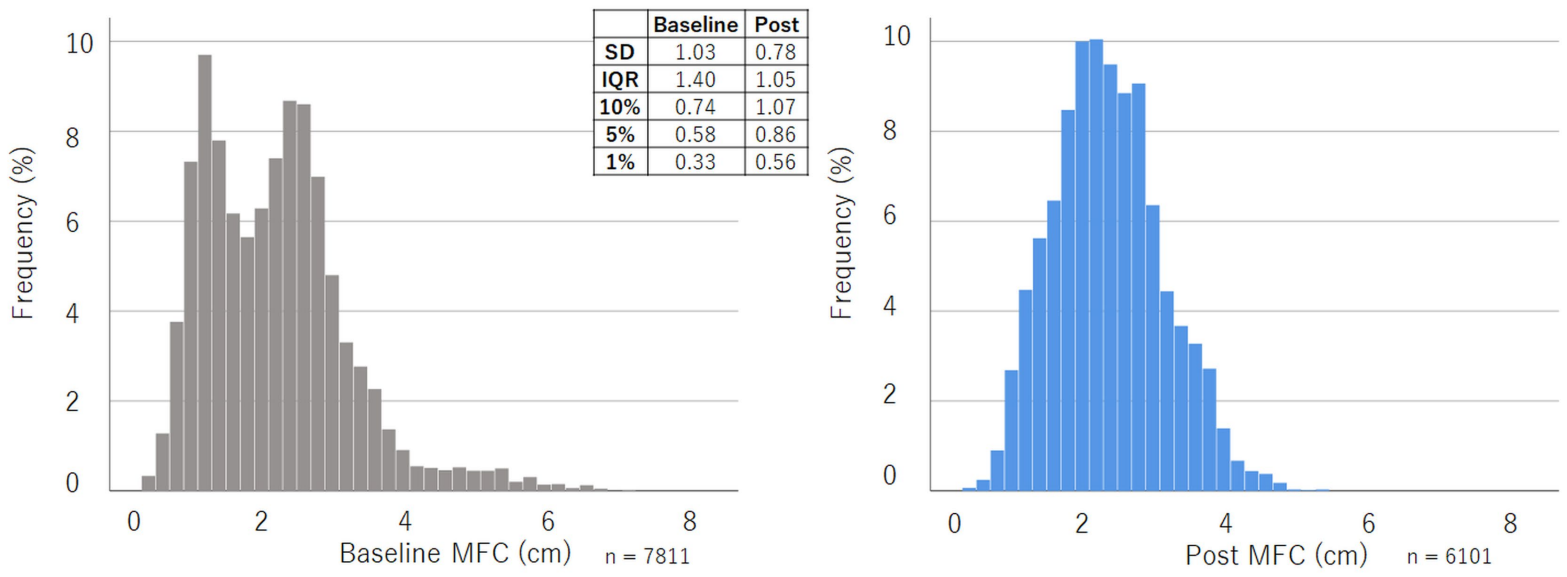
Histogram characteristics of MFC (left) Baseline, (right) Post HAL training, units used in the table were all centimeter (cm).

Histogram descriptions incorporating all steps from every subject were combined and displayed in Figure 3 to visualize collective MFC characteristics (12 participants, 7,811 MFC for Baseline/6,101 MFC for Post training). The discrepant number of MFC values resulted from marker-occlusion-based exclusions, including removal of the corresponding paired contralateral steps. Variability measures (SD, IQR) and lower percentiles (10, 5, 1%) were obtained for the combined data from all participants and both limbs, revealing differences compared to the averaged values in Table 1. Figure 3 shows that lower-end percentiles generally increased, with reduced variability in the Post condition. Additionally, the upper-range distribution appeared to diminish. Compared to the Baseline, Post-training may have resulted in lower inter-participant differences in MFC patterns.

## 4 Discussion

As the extension of our previous study (Nagano et al., 2022a), we explored the training effects of active ankle exoskeleton on swing foot clearance and tripping risk. The results suggest that just 5 min of ankle training altered overall MFC distribution characteristics associated with the multivariate training main effect supported by a large effect-size estimate. As shown in Figure 3, both the higher and lower extremes were reduced, and the dataset became more concentrated, even when combining multiple participants’ data. By optimizing MFC control, both insufficient and exaggerated ankle motion were eliminated. Among healthy individuals, this could result in optimal MFC characteristics (Figure 3).

Further statistical analysis supports the positive impact on MFC, including reduced step-to-step variability, increased skewness, and an elevated lower end (1%), with these follow-up outcomes accompanied by medium effect-size estimates (Begg et al., 2007; Best and Begg, 2008; Mills et al., 2008; Nagano et al., 2022b). Notably, the 5-min training module did not affect the mean or median values but significantly increased the lower percentile (1%), which is crucial for reducing tripping risk (Nagano et al., 2022b). The increase in the 1% value reflects the elimination of the lower extremes in the MFC dataset (Figure 3). We have previously tested a variety of interventions to increase MFC, such as specially designed insoles, biofeedback gait retraining for stroke survivors, and exercise intervention for frail older adults (Tirosh et al., 2013; Nagano and Begg, 2021; Sarashina et al., 2022). However, it remains unclear whether these previous methods improved the lower percentiles like the current exoskeleton-based intervention, which is the key to reducing tripping risk.

The reduction in MFC variability is a well-recognized adaptation for minimizing tripping risk (Begg et al., 2007; Mills et al., 2008). Consistent with our previous pilot work (Nagano et al., 2022a), the current results demonstrated that the present exoskeleton-based neuromotor training approach improved the consistency of ankle movement control, achieving a notable reduction in MFC variability after only a 5 min-engagement. This is likely due to more stable tibialis anterior activity during ankle-focused exoskeleton training (Kubota et al., 2022; Matsuda et al., 2022), supporting the view that consistent dorsiflexion control across steps reduces variability. Neuromotor training is typically more difficult to precisely target than isolated muscle strengthening, yet the current method appeared to facilitate correct and repeatable movement patterns, possibly through enhanced sensorimotor integration.

Active exoskeletons of this type can be applied in various neurological conditions. Such systems utilize sEMG signals, which are processed through filtering and amplification to extract features relevant for intention-based control. As a result, even individuals with neuromuscular impairments can repeatedly engage in intention–movement coupling, providing interactive biofeedback that may reinforce neural circuits through concurrent afferent and efferent stimulation (Kubota et al., 2022; Matsuda et al., 2022; Soma et al., 2021). In contrast, walking in healthy adults is largely automatic. Therefore, seated ankle training using the exoskeleton likely supported more consistent ankle kinematics and MFC patterns not by explicit real-time control, but by repeatedly guiding individuals through a defined range of motion. This may promote more stable neuromotor patterns that subsequently manifest during gait, even without conscious regulation. However, because the training was performed in a seated position, the protocol did not reproduce the task-specific demands of active treadmill walking, including body-weight support, dynamic balance control, and phase-dependent ankle control during gait. Therefore, the observed post-training changes should be interpreted as acute transfer from isolated ankle-movement practice to walking, rather than as evidence that seated training directly optimizes gait-specific control.

In highly repetitive motor behaviors such as walking, step-to-step variability partly arises from noise within the sensorimotor system, including fluctuations in motor unit recruitment, variability in supraspinal–spinal integration, and imperfect matching of neural commands to the executed movement (Faisal et al., 2008; Farina and Negro, 2015). Such variability can manifest as subtle and unpredictable micro-oscillations in ankle motion output, influencing swing-foot clearance. Training approaches that repeatedly align voluntary neural drive with the resulting ankle motion may help transiently refine sensorimotor integration by improving the correspondence between intended and executed movements. Although the present study cannot confirm neural adaptations directly, intention–movement coupling has been proposed as a mechanism that may strengthen task-relevant circuits, potentially improving the efficiency or consistency of neural signaling over repeated cycles of practice. These short-term effects do not imply structural regeneration, but they are consistent with the concept of rapid neural tuning observed in other motor learning contexts (Leech et al., 2022).

Future studies should investigate effects in vulnerable populations who may benefit most from reduced tripping risk (Fasano et al., 2017; Killeen et al., 2017), as this pilot study included only healthy adults. The treadmill protocol at 4 km/h eliminated speed-dependent influences on MFC, while overground walking at preferred speeds may produce different outcomes. Neuromotor training was limited to a single 5-min session with an assist level of 20 (out of 1,200), and it remains unclear whether longer training durations or alternative assist levels would produce stronger or more sustainable effects. Future work should also investigate how these effects persist over time, particularly because longer-term ankle robotics training has been reported to produce retained gait-biomechanical improvements, including toe-clearance-related outcomes (Roy et al., 2026). The ankle exoskeleton used in this study (HAL) operates through intention-based control using sEMG signals. However, whether the observed effects require this specific mechanism remains unknown. Other active exoskeleton devices—such as systems driven by inertial sensing, impedance-based control, or externally paced actuation—may produce comparable or even superior outcomes (He et al., 2024). Therefore, the present findings should be interpreted as preliminary proof-of-concept evidence that brief intention-based ankle exoskeleton training can acutely alter MFC distribution characteristics. Future studies should include appropriate control conditions, such as sham assistance or time-matched no-exoskeleton protocols, to determine whether the observed changes are attributable specifically to intention-based ankle exoskeleton training.

A further challenge is the lack of standardized MFC measurement protocols, which complicates inter-study comparisons due to differences in footwear, marker configurations, and walking environments, as noted by Al Bochi et al. (2021). Nonetheless, within-setup comparisons remain valid. Despite symmetry assessments, no limb differences in MFC were observed in this study; however, gait asymmetry is common in vulnerable groups such as older adults and stroke survivors (Alexander et al., 2009). Exoskeleton-based ankle training may support coordinated, symmetrical movement patterns, although this requires confirmation in asymmetrical populations.

Focusing on the lower tail (1%) of the MFC distribution was essential, as no differences were detected in mean or median values, and clinically meaningful effects would have been overlooked. This analysis allows a clearer redefinition of “safe” MFC characteristics. Although high swing foot clearance is important to prevent tripping, the assumption that “higher is always safer” may be misleading. Raising the lower end of the distribution is likely more critical. In other words, critically low MFC values should be avoided, and first-percentile measures may provide a more accurate indicator of tripping risk than central-tendency metrics alone (Nagano et al., 2022b).

As noted in previous studies (Begg et al., 2007; Best and Begg, 2008; Mills et al., 2008), low variability in swing foot clearance is important for minimizing irregular MFC control. If the focus is on the lower end, the interquartile range (IQR)—which reflects the spread between the 25th percentile and the 75th—may not fully capture the variability that matters clinically. Future studies could consider alternative metrics such as standard deviation (SD) or adapted coefficients of variation (e.g., SD divided by the first percentile) to evaluate MFC variability in lower percentiles.

## 5 Conclusion

Ankle exoskeleton training controlled by the wearer’s own intention may help reduce the occurrence of critically low Minimum Foot Clearance (MFC) values within the lowest percentiles of the distribution, which are closely associated with increased tripping risk. This study highlights the importance of focusing on lower MFC percentiles, as changes in the lowest end of the distribution provide a more accurate indication of tripping risk than central-tendency measures alone. Future research should examine longer-term adaptations and evaluate this approach in higher-risk populations, including older adults and individuals with neuromotor impairments. Larger controlled studies will be required to determine whether the present acute findings translate to meaningful reductions in tripping risk.

## Data Availability

The original contributions presented in the study are included in the article/supplementary material, further inquiries can be directed to the corresponding author.
